# Cluster randomized controlled trial protocol: addressing reproductive coercion in health settings (ARCHES)

**DOI:** 10.1186/s12905-015-0216-z

**Published:** 2015-08-06

**Authors:** Daniel J. Tancredi, Jay G. Silverman, Michele R. Decker, Heather L. McCauley, Heather A. Anderson, Kelley A. Jones, Samantha Ciaravino, Angela Hicks, Claire Raible, Sarah Zelazny, Lisa James, Elizabeth Miller

**Affiliations:** UC Davis Department of Pediatrics and Center for Healthcare Policy and Research, 2103 Stockton Blvd Suite 2224, Sacramento, CA 95817 USA; Division of Global Public Health in the Department of Medicine & Center on Gender Equity and Health, University of California, San Diego, 9500 Gilman Drive #0507, La Jolla, CA 92093-0507 USA; Department of Population, Family and Reproductive Health, Johns Hopkins Bloomberg School of Public Health, 615 N. Wolfe Street, E4142, Baltimore, MD 21205 USA; Department of Pediatrics, Division of Adolescent Medicine, University of Pittsburgh School of Medicine, Children’s Hospital of Pittsburgh of UPMC, 3420 Fifth Ave, Pittsburgh, PA 15213 USA; Futures Without Violence, 100 Montgomery Street, The Presidio, San Francisco, CA 94129 USA

## Abstract

**Background:**

Women ages 16–29 utilizing family planning clinics for medical services experience higher rates of intimate partner violence (IPV) and reproductive coercion (RC) than their same-age peers, increasing risk for unintended pregnancy and related poor reproductive health outcomes. Brief interventions integrated into routine family planning care have shown promise in reducing risk for RC, but longer-term intervention effects on partner violence victimization, RC, and unintended pregnancy have not been examined.

**Methods/Design:**

The ‘Addressing Reproductive Coercion in Health Settings (ARCHES)’ Intervention Study is a cluster randomized controlled trial evaluating the effectiveness of a brief, clinician-delivered universal education and counseling intervention to reduce IPV, RC and unintended pregnancy compared to standard-of-care in family planning clinic settings. The ARCHES intervention was refined based on formative research. Twenty five family planning clinics were randomized (in 17 clusters) to either a three hour training for all family planning clinic staff on how to deliver the ARCHES intervention or to a standard-of-care control condition. All women ages 16–29 seeking care in these family planning clinics were eligible to participate. Consenting clients use laptop computers to answer survey questions immediately prior to their clinic visit, a brief exit survey immediately after the clinic visit, a first follow up survey 12–20 weeks after the baseline visit (T2), and a final survey 12 months after the baseline (T3). Medical record chart review provides additional data about IPV and RC assessment and disclosure, sexual and reproductive health diagnoses, and health care utilization. Of 4009 women approached and determined to be eligible based on age (16–29 years old), 3687 (92 % participation) completed the baseline survey and were included in the sample.

**Discussion:**

The ARCHES Intervention Study is a community-partnered study designed to provide arigorous assessment of the short (3-4 months) and long-term (12 months) effects of a brief, clinician-delivered universal education and counseling intervention to reduce IPC, RC and unintended pregnancy in family planning clinic settings. The trial features a cluster randomized controlled trial design, a comprehensive data collection schedule and a large sample size with excellent retention.

**Trial Registration:**

ClinicialTrials.gov NCT01459458. Registered 10 October 2011.

## Background

Women ages 16–29 years seeking care in family planning (FP) clinics report higher rates of intimate partner violence (IPV) compared to their same-age peers; [1–7] these negative experiences are, in turn, associated with unintended pregnancy and related poor sexual and reproductive health outcomes [8–19]. A growing body of literature on male partner influences on contraception and pregnancy decision-making has identified a range of male partner pregnancy-controlling behaviors that contribute to poor reproductive and sexual health outcomes for women [7, 20–23]. Reproductive coercion (RC) is defined as male partners’ attempts to promote pregnancy in their female partners through verbal pressure and threats (pregnancy pressure), direct interference with contraception (birth-control sabotage), or threats and violence related to pregnancy continuation or termination (control of pregnancy outcomes) [1, 7, 24, 25]. In our pilot intervention study, 53 % of young women using FP clinics reported ever experiencing IPV, and 25 % reported reproductive coercion, the combination of which was strongly associated with increased prevalence of unintended pregnancy [26].

In light of increased sexual and reproductive health concerns and care utilization among IPV victims, as well as the success of clinic-based interventions regarding other behaviorally-based health issues, [27–32] multiple calls for clinic-based interventions for IPV have emerged [33–38]. Unfortunately, screening and disclosure rates remain low; existing studies on disclosure indicate that only 5 % to 15 % of women have disclosed abuse to their providers [39–41]. Survivors’ preferences not to be pressured to disclose [42–44] and health provider discomfort with discussing IPV [45–47] compound the difficulties in identifying and supporting survivors [34, 44, 48–55]. IPV screening alone, in the absence of counseling interventions, has not been found to be effective in reducing IPV or other related health outcomes for women [56, 57]. Thus, interventions that provide information and support for all women seeking clinical care and facilitate routine discussion of IPV within the clinical context are indicated [58, 59]. Evidence that abused women also face reproductive coercion (RC) suggests the promise of integrating discussion of RC within IPV assessment to facilitate women’s recognition of these interrelated issues. To our knowledge, the ARCHES intervention is the first to include formal assessment of RC for women seeking reproductive health services and to facilitate patient and provider discussion of RC and related abusive experiences.

The ARCHES (Addressing Reproductive Coercion in Health Settings) intervention was developed collaboratively by researchers, advocates and community-based practitioners, with significant input from FP clients [26]. Providers are trained to conduct the three components of the ARCHES intervention: 1) universal client education and assessment regarding IPV and RC; 2) discussion of harm reduction behaviors to reduce risk for unintended pregnancy, IPV victimization, and RC; 3) supported referrals to IPV victim services (including provision of IPV related resources to all clients regardless of disclosure). ARCHES is designed to be implemented within routine FP care, maximizing its feasibility, sustainability and scalability. In the initial pilot randomized intervention trial, among women who at baseline reported experiencing IPV in the past 3-months, the original version of ARCHES reduced the odds of recent pregnancy coercion (a component of RC) at follow-up assessment (3 to 6 months post-baseline) by 71 % compared to the control, highlighting the potential impact of this intervention [26]. Based on this pilot, we designed a large-scale cluster randomized controlled clinical trial (RCT) (i.e., sufficiently powered and of adequate duration) to assess the short (3–4 month) and long-term (12 month) effects of this innovative program on IPV and RC (primary outcomes) as well as unintended pregnancy (secondary outcome) -- major and prevalent health threats among medically underserved women.

ARCHES is being evaluated in 25 FP clinics in Western Pennsylvania randomized to either intervention or control (i.e., standard-of-care) conditions. Participating female FP clients ages 16–29 (*N* = 3687) complete assessments at baseline (T1), 12–20 weeks (T2), and 12 months (T3) to assess intervention effects on knowledge and behaviors related to IPV, RC and related harm reduction, as well as unintended pregnancy. Survey data at each time point are collected via audio computer assisted self-interview in English or Spanish. Chart data abstracted from client medical records allow tracking of clinic utilization, pregnancy testing, and diagnosed pregnancies one year prior to baseline through 18 months post-baseline. Intervention effects on patient-level outcomes at follow-up will be assessed using multilevel regression analyses that account for clustering of individual participants within clinics, the units of randomization. All project partners, including client and clinician advisors, participate in interpretation of results and dissemination of findings.

## Methods/Design

### Overview of RCT design

**Fig. 1:**
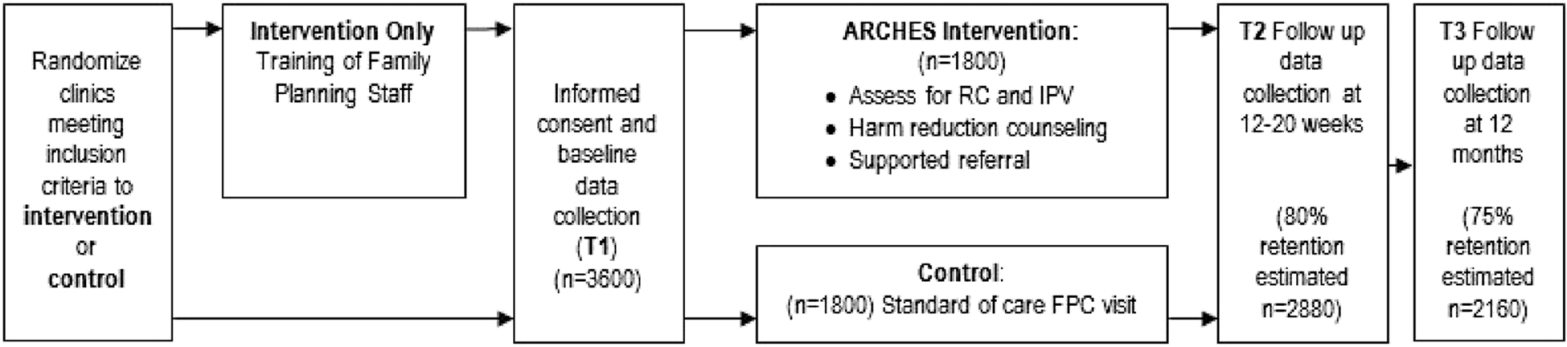
Study design, including timing of assessments and targeted enrollment. Patients in this cluster randomized trial are scheduled for assessments at three timepoints. The targeted enrollment was based on power calculations with cautious assumptions regarding patient retention at follow-up timepoints

The ARCHES Intervention Study was designed as a stratified, parallel-group cluster randomized controlled trial comparing the ARCHES intervention to standard-of-care for family planning clients (Fig. 1 and Table 1). Because ARCHES is designed to facilitate changes in clinical culture related to how abuse is handled, a cluster randomized design was required to avoid contamination that could arise from within-cluster randomization of patients or providers. Family planning clinics were classified as rural or urban location based on U.S. Census and Federal Register data from the Bureau of Commerce [60], grouped into clusters based on shared providers, stratified by cluster size and then randomly assigned either to receive provider training on the ARCHES intervention and implement this with all clinic visits or to continue their usual assessment and care for domestic violence. Randomization provided each clinic an equal probability of being in either arm. To ensure balance, randomizations resulting in a between-arm imbalance in the number of rural clinics of 5 or greater were not allowed.

**Table 1:** Outcome measures and data collection points

Outcomes	Participant survey measurement points				Chart Abstraction Data	Measures
	T1		T2	T3		
	Prior to clinical visit	Immediately after clinic visit	12–20 weeks	1 year	12 months pre-baseline to 18 months post-baseline	
Primary Outcomes						
Recent reproductive coercion	X		X	X		10 items, investigator-developed (summary score)
Recent physical and sexual partner violence victimization	X		X	X		Conflict Tactics Scale-2 (CTS2; 1 item on physical violence) [91]
						Sexual Experiences Survey (2 items on sexual violence) [86] (summary score)
Secondary Outcomes						
Unintended pregnancy^a^	X			X	X	Self-report 7 items from National Survey of Family Growth (summary score); electronic medical record data^1^
Recognition of sexual and reproductive coercion	X		X	X		9 items, investigator-developed (mean score)
Self-efficacy to implement harm reduction strategies			X	X		4 items, investigator-developed (mean score)
Knowledge of IPV-related resources and services			X	X		5 item checklist of national and local resources (summary score)
Use of harm reduction strategies			X	X		6 items, investigator-developed (summary score)
Additional Secondary Outcomes *(restricted to those reporting physical or sexual partner violence or RC at baseline)*						
Use of IPV-related resources and services			X	X		5 item checklist of national and local resources (summary score)
Disclosure to health care provider Reproductive coercion				X		Two items on telling HCP about IPV or RC experiences
Intervention Implementation *(to assess intensity of intervention received for as-treated analyses)*						
Conversation with HCP about healthy and unhealthy relationships		X				1 item, self-report
Receipt of safety card		X				1 item, self-report

^a^Information is assessed both via self-report and medical record data, such that the greater value of the two data sources is used for analyses

**Fig. 2:**
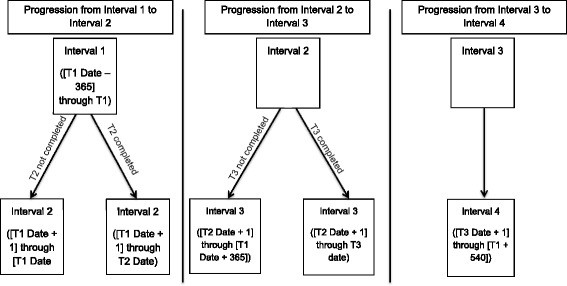
Definition of intervals for use in electronic medical record abstraction. To match the reporting periods spanned by the T1, T2 and T3 surveys, events identified in electronic medical record (EMR) data are assigned to patient-specific intervals using the displayed definitions

Patients in both arms complete surveys at baseline prior to their clinical visit, immediately following their clinical visit, 12–20 week follow-up, and 1 year follow-up (Fig. 1). Surveys are completed using laptops with audio computer-assisted self-interview (ACASI) at baseline; follow-up surveys are also completed via ACASI (for clients able to come to clinic) or via an online survey sent via email (for 18 years of age or older) or by telephone for women unable to return to the clinic. Following completion of survey data collection, electronic medical record (EMR) data are abstracted for defined intervals within the time period one year prior to baseline and up to 18 months post baseline (Fig. 2).

### Description of intervention

#### Relevant rationale, history and format of the ARCHES intervention

**Fig. 3:**
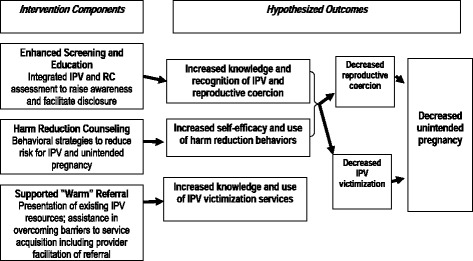
Conceptual model for ARCHES

This study evaluates a family planning clinic-based IPV and reproductive coercion (RC) intervention developed by a team of researchers, victim service advocates, and reproductive health practitioners [61]. Figure 3 describes the conceptual model of the ARCHES Intervention and Hypothesized Outcomes. One innovation of the ARCHES intervention is the focus on training not only clinicians, but also para-medical providers (i.e., medical assistants, health educators, and family planning counselors working in these settings) to discuss IPV and RC when counseling around contraception, pregnancy or STI testing. Additionally, the emphasis on universal provision of IPV/RC information recognizes that women often do not recognize IPV or RC and may not define sexual coercion as abuse, [62, 63] particularly when the perpetrator is known to them [63]. The lack of recognition of abusive behaviors in relationships [42, 43] has been associated with decreased IPV help-seeking, [63–65] highlighting need for universal IPV/RC education and enhanced assessment.

ARCHES provides universal IPV/RC education and enhanced assessment through FP provider discussion of IPV/RC with their patients in a way that highlights the prevalence of such abuse among women seen at the clinic and educates patients about the reproductive health impact of such abuse. The enhanced assessment for IPV/RC integrates into the reproductive health visit, for example, by asking a woman seeking pregnancy testing whether her partner might be pressuring her to get pregnant. This education and assessment is facilitated by the use of a palm-sized brochure which describes healthy and unhealthy relationships, offers information about harm reduction, and provides IPV related resources. Evidence that clinic-based IPV assessment can be the first step in recognizing abuse, particularly when done in a context that normalizes such abuse experiences, [42, 66] strengthens the rationale for locating IPV and RC assessment within the context of supportive education for all women seeking FP services.

ARCHES also counsels women on harm reduction strategies. Harm reduction, originally used within substance abuse treatment, has been effective in managing a range of health risk behaviors [67] by ‘meeting clients where they are’ and assisting them with identifying strategies to decrease harm, including harms related to sexual health [68–71]. IPV interventions appear to increase safety planning and harm reduction behaviors among victimized women, e.g., increase ability to refuse sex, [72] reduce substance use in dating contexts, [73] and advance preparation for safe escape should violence escalate [74]. Harm reduction behaviors have also been shown to protect against violence victimization among high-risk groups (i.e., women in prostitution), [75] and to reduce revictimization among college women [73]. Thus, ARCHES is designed to reduce women’s risk for violence victimization and unintended pregnancy via education regarding non-partner dependent contraceptives (longer acting reversible contraceptives such as the intrauterine device), access to emergency contraception, and provision of harm reduction strategies.

Finally, supported “warm” referrals (i.e., provider facilitation of referral to a victim service advocate via phone or in person) can assist clients in overcoming common barriers to accessing services, including self-blame, [43, 76] lack of recognition of abuse, [63, 64] lack of knowledge of services, [43, 76, 77] and perception that services are limited in scope (e.g., solely crisis oriented) [76]. Articulating the scope of services available to all women (regardless of disclosure of IPV or RC experiences), and normalizing use of these services may facilitate awareness and use of IPV services, improve mental health symptoms, [78–81] and reduce revictimization [82–84].

#### Training of intervention sites

Nine clusters (11 clinics) were randomized to the intervention. In these clinics, family planning clinic staff (family planning counselors, medical assistants, and clinicians) were trained to educate clients about the links between reproductive health concerns and intimate partner violence/sexual assault (“enhanced screening”); counsel clients on harm reduction behaviors to reduce risk for IPV/RC (“harm reduction”); and provide clients with information on violence victimization support services (“supported ‘warm’ referrals”) with the goal of reducing IPV/RC and unintended pregnancy. The training, led by one of the PIs and the lead trainer from Futures without Violence (the non-profit violence prevention organization collaborating on this study), involved a half-day (approximately 3 h) introduction on the prevalence of IPV/RC, instruction on how to conduct universal IPV/RC client education with the aid of the palm-sized brochure, and role playing to practice IPV/RC assessment and provide harm reduction counseling for specific clinical scenarios (such as pregnancy testing). Training materials included copies of the reproductive health palm-sized brochure, reproductive health care setting medical guidelines, slides describing the prevalence of IPV and RC and relevance for family planning services, and video vignettes illustrating common reproductive health visits demonstrating with actors how to implement this universal education and brief counseling intervention.

Approximately 1–3 months after the initial training, in conjunction with starting data collection, a 30-minute refresher training was conducted by the study coordinator, to review key points of the training and provide opportunity for providers to ask clarifying questions about the intervention to increase fidelity.

#### Assessing fidelity to intervention

To maintain quality of intervention delivery and track fidelity to the intervention, data collected from client exit surveys (brief surveys completed by clients as soon as their clinic visit was over) were summarized on a weekly basis, and sent via email to the office manager of each intervention site. The data highlighted client responses to two questions: “Today, did your health care provider talk with you about healthy and unhealthy relationships?” and “Today, did your health care provider give you a card called” Do You Know Your Relationship Affects Your Health?“talking about partner’s control of pregnancy and your reproductive health?” The proportion of clients reporting they had had this discussion and had received the palm-sized brochure was reported weekly to each office manager, with the study coordinator offering suggestions on strategies to increase provider discussions and card delivery.

#### Control condition

Eight clusters (14 clinics) were randomized to the standard-of-care (‘control’) condition, meaning they continued diagnostic and treatment procedures as clinic staff typically would for their patients with no additional training. In discussions of sexual health or pregnancy risk, IPV may be discussed, but no training on or provision of protocols related to IPV education or referral were included as standard-of-care. Apart from such standard care, at the conclusion of the exit survey at the baseline clinic visit, control group participants received written materials regarding community resources which included IPV resources mixed in with other information around housing, food, and social services. Assigned to a wait-list condition, a control site could opt to receive the ARCHES intervention training following the completion of follow-up data collection.

### Outcome measures

#### Primary outcomes

##### Reproductive coercion

Recent (past 3 months) reproductive coercion, specifically, pregnancy pressure and birth control sabotage, is assessed using a scale of 10 items (e.g., Has someone you were dating or going out with: “said he would leave you if you didn’t get pregnant” and “made you have sex without a condom so you would get pregnant” (Cronbach alpha = 0.74)) [1, 7]. A summary score tallies the number of behavior items experienced, for a maximum score of 10.

##### Recent physical and sexual partner violence victimization

Past three month physical and sexual partner violence are measured using one item modified from the Conflict Tactics Scales-2 [85] for physical violence (i.e., hit, pushed, slapped, choked or otherwise physically hurt by someone they were dating or going out with) and two items from the Sexual Experiences Survey [86] for sexual violence (being made to do something sexual with and without the use of force or threats) (Cronbach alpha = 0.46). The outcome is modeled as a summary score (maximum value of 3 if a participant experienced all 3 actions).

#### Secondary outcomes

##### Incident and unintended pregnancy

Incident pregnancies are measured using two methods: a single survey item asking how many times the client had been pregnant in the past 12 months, including miscarriages and abortions, and chart abstraction data indicating a positive pregnancy test, not receiving a pregnancy test because patient is currently pregnant, or receiving prenatal care. The greater of self-reported pregnancies or chart abstraction data on pregnancy diagnoses is counted as the total number of incident pregnancies for that client in each time interval (12 months prior to baseline and 18 months after baseline).

Self-reported unintended pregnancy is measured via 7 items from the National Survey for Family Growth, as recommended by Santelli and colleagues to assess pregnancy intention (i.e., desire and timing) [87]. Women who reported any pregnancy in the past 12 months are asked, for their most recent pregnancy, three dichotomous items about the timing (mistimed), planning (unplanned), and desire to have a baby with their current partner (not desired). Four scaled items ask about how much they wanted to be pregnant (“wanted to avoid” to “wanted to get pregnant”), how much they were trying to get pregnant (“not trying” to “really trying hard”), trying to avoid getting pregnant (“trying to avoid” to “not trying to avoid”), and how happy they were when they found out they were pregnant (“very unhappy” to “very happy”). For each of these four items, the scale is from zero to 4, with responses of zero and 1 coded as unintended. In multivariate analyses, these 7 items were unidimensional [88]. Thus, a summary score from responses to all 7 items is created to measure the degree of unintendedness of the pregnancy, ranging from 1 to 7 (Cronbach alpha = 0.94). Women with no pregnancy in the past year and women who had been pregnant but have no ‘unintended’ responses to the above 7 items are coded as zero (i.e., no unintended pregnancy).

#### Recognition of sexual and reproductive coercion

A scale of 9 items was developed by the investigators to assess participants’ perceptions of how abusive behaviors specific to sexual and reproductive coercion are (e.g., “trying to make someone have an abortion when they don’t want one” and “pressuring someone to have sex when they’ve said no”). Participants are asked to rate the behaviors on a 4-point Likert scale from “not abusive” to “extremely abusive” (Cronbach alpha = 0.86). The outcome is measured as a mean score of the 9 responses with a higher score indicating greater perception of abusiveness.

#### Self-efficacy to implement harm reduction strategies

Participants are presented with four statements that are designed to assess level of confidence in implementing behaviors to reduce the impact of reproductive and sexual coercion (e.g., “If my sexual partner is keeping me from using birth control, I am confident I could ask a healthcare provider about other kinds of birth control that my partner can’t mess with.”). Responses are rated on a 5-point Likert scale from “strongly agree” to “strongly disagree” with the outcome modeled as a mean response to the 4 items (Cronbach alpha = 0.72). To reduce measurement effect (i.e., the survey items themselves increasing women’s knowledge of harm reduction strategies), this construct is assessed at follow-up visits only (T2 and T3).

We also collected a general self-efficacy measurement at baseline, using The New General Self-Efficacy scale (NGES) developed by Chen, Gully, and Eden [89] to acquire the statistical power-enhancing advantages of a baseline covariate that is correlated with the above self-efficacy outcome, but whose assessment would not lead to a “measurement effect.” The NGES consists of 8 items (e.g., “Even when things are tough, I can perform quite well.”) with response options on a 5 point Likert scale (“strongly agree” to “strongly disagree”) (Cronbach alpha = 0.89). General self-efficacy is modeled as the mean response to these items and the baseline value is included as a covariate in the models assessing self-efficacy to implement harm reduction strategies.

#### Use of harm reduction strategies

Use of 6 harm reduction strategies to decrease the occurrence and impact of reproductive and sexual coercion and unsafe relationships was assessed at both follow-up visits. Participants are asked to endorse whether they had used these strategies (e.g., “have you hidden your birth control from your partner because you were afraid he would get upset with you for using it” and “have you tried to protect yourself from your partner by asking family or friends to call or text you when you were with that partner”) in the past 3 months (Cronbach alpha = 0.39). The outcome is modeled as a summary score with a maximum value of 6 (one point per each strategy endorsed). To prevent measurement bias, this construct is assessed at follow-up visits only (T2 and T3).

#### Knowledge of IPV-related resources and services

To assess knowledge of IPV-related resources and services, participants are presented a list of resources and asked to endorse each one for which they “had received information on in the past 3 months” (Cronbach alpha = 0.80). Listed resources included Domestic Violence Advocacy Services, Rape Crisis Center, National Domestic Violence Hotline, Teen Dating Abuse Helpline, and Loveisrespect.org. A summary score is calculated with one point per resource selected for a maximum score of 5. To reduce the possibility of participants endorsing items because they had seen the resources identified in earlier surveys, this measure was not included in the baseline survey.

#### Additional secondary outcomes for women with recent history of IPV or RC

In addition to the above outcomes, two additional outcomes are included for analyses restricted to women who endorsed any physical or sexual violence or RC at baseline, as this group was hypothesized to receive the greatest benefit from the intervention. For convenience, we refer to this analysis subset as the “Aim 2” analysis, with the full population analyses described as “Aim 1” analyses.

#### Use of IPV-related resources and services

Participants are asked to endorse all IPV-related resources that they used in the past 3 months from the same list presented in the measure of knowledge of IPV-related resources and services (above) (Cronbach alpha = 0.55). As with the knowledge measure, this question is presented at follow-up visits only (T2 and T3). The outcome is modeled as a summary score, tallying number of endorsed items.

*Disclosure to a health care provider* was assessed at T3 only with two questions: whether in the past 12 months the client told a health care provider about ever being in an unhealthy or unsafe relationship or about a partner trying to get her pregnant when she didn’t want to be, messing with birth control, or controlling a pregnancy.

### Setting

Patients were recruited from 25 family planning clinics affiliated with Planned Parenthood of Western Pennsylvania and Adagio Health from both urban and rural areas within a 150 mile (3 h) radius of Pittsburgh, Pennsylvania. All were free-standing family planning clinics; family planning services embedded within community health centers were excluded. Some clinics were combined into clusters (*n* = 17 clusters) to account for shared staff and providers that could otherwise lead to contamination between the two arms of the trial.

### Randomization procedures, including choice of unit of randomization

The unit of randomization was clinic clusters comprised of one or a small number of clinics sharing a common set of healthcare providers. We chose a clinic-level cluster-randomized design to prevent contamination of the control condition (standard of care). Given that a main component of this intervention was training health care providers how to facilitate conversations with patients about threats to reproductive and sexual health (such as RC and IPV), it would not be feasible or appropriate to request a provider to initiate conversations with only a subset of patients, which would be the case if the study had used individual client-level randomization. A second component of the intervention was to connect clinics to domestic violence advocates to facilitate referrals to community violence resources; this clinic-level component precluded the use of provider-level randomization. The study was also intended to assess the effectiveness of this intervention in “real world” clinical settings, i.e., with expected variability in staff and provider comfort in addressing IPV and RC and implementation of the intervention.

To ensure balanced assignment by clinic setting, each cluster was labeled as urban or rural based on Census and Department of Commerce Federal Register data [60]. Clinics with common providers were clustered together. Randomization was stratified by cluster size and was designed to give each cluster an even probability of being randomized to intervention or control condition. Nine of the 10 single-clinic clusters were rural, with five randomized to treatment. Of the 6 two-clinic clusters, 2 were rural, 1 was urban and 3 were mixed. The randomization assigned the three mixed clinics to the control condition. The single 3-clinic cluster was all rural. It was assigned to the control condition, as well. Had the randomization resulted in a between-arm imbalance of 5 or greater rural clinics (a 16.25 % probability), it would have been discarded and a new randomization performed. Randomization was performed using SAS software by the study’s lead statistician (DJT).

### Participants: eligibility, recruitment and institutional review board requirements

#### Description of IRB approval

The study was approved by the University of Pittsburgh Institutional Review Board (IRB) on June 21, 2011 by expedited review (Protocol Number PRO11050458). The University’s IRB approval included a waiver for the requirement to obtain a written informed consent to participate in the research, in order to reduce the risk for breach of confidentiality (considered the primary risk associated with this study); women were offered a copy of an information sheet that was reviewed verbally with all clients. For minors ages 16 and 17, a waiver of parental informed consent for research participation was also approved by the IRB to protect youth seeking confidential services at these clinics from breach of confidentiality. A federal Certificate of Confidentiality was also obtained for the study to protect research records from subpoena.

Additional safety precautions were in place to reduce risk of harm to women for participating in the study, consistent with international recommendations and best practices on violence-related research [90]. Women who were at the clinic with a male partner were only approached about the study when they were alone. All follow up contacts made about the study referred to the study as the “Women’s Health Study.” To mitigate risk for emotional distress potentially associated with taking a survey about IPV, all research assistants were trained to ask women about how they were feeling after taking the survey, offer a list of community resources (with IPV and sexual assault information embedded among other less sensitive information about housing and food), and to suggest women speak with the clinical staff on site if they wished to talk more about the survey questions. Additionally, if any woman disclosed to a research assistant during survey administration that she was concerned about her safety, research assistants were trained to connect women with clinical staff as well as the local victim service advocacy organization. Finally, for follow up surveys conducted via email, women were encouraged to find a computer in a private space to complete the survey and to erase the history after survey completion. For those completing a follow up survey by phone, the research coordinator would suggest a special ‘code phrase’ the woman could use if she needed to hang up because someone else could overhear the conversation.

#### Procedures on Day of clinic visit

Research assistants (RAs) approached women in the waiting room of FP clinics after they checked in for their clinic visit if they were not in the presence of a male partner and asked if they were here for a clinic visit today, and whether they would like to hear about a women’s health research study. If they were interested, the RA assessed for eligibility. If eligible, potential participants were brought to a private space in the clinic to complete the consent process and survey administration. RAs let clinic staff know that the patient may be interested in being in the study and would be taking the survey before her appointment if she consented.

RAs explained to potential participants that they would complete two surveys (one before and one immediately after their clinic visit today) and receive a $15 gift card, one survey in approximately 3 months for $25, and one survey in 12 months for $40 to thank them for their time and effort.

#### Prior to clinical encounter

Before participants’ appointments, they provided their contact information (phone number and email address) and two alternate contacts to the RA. RAs also went over the verbal consent sheet, which provided details on why the study was being conducted, the data collection process at each of the three time points, the implications of the Certificate of Confidentiality that was obtained for the study, the voluntary nature of participating, and the schedule of compensation. RAs then answered any questions participants had and asked participants to sign an optional medical chart release form (provided by the clinic) for chart abstraction at the conclusion of the study. After being assigned a four-digit ID number for the survey data, participants completed an audio computer-assisted survey instrument (ACASI) on a laptop with the option to have questions read aloud (in English or Spanish) through headphones. When participants finished with the baseline survey, they either returned to the waiting room to wait to be seen by the clinician(s) or went directly to an exam room for their scheduled clinical visit. RAs communicated with clinic staff to indicate which participants had taken the baseline survey so that they could direct the patients (after their clinical visits) back to the RAs for the exit survey.

#### Following clinical encounter

After the participants’ clinic visits, they were asked to return to the private room with the RA to complete the client exit survey, also completed using the ACASI program on a laptop. Upon finishing this survey, RAs issued $15 gift cards, local resource sheets, and appointment reminder cards for the next follow survey to all participants. In intervention clinics, RAs also offered extra palm-sized brochures to participants, explaining one was to keep, and one was to give away to either a friend or family member. Control clinic participants only received the one page community resource sheet. When distributing brochures and resource sheets to participants, RAs told participants that taking these materials was voluntary, if they felt safe taking the materials with them.

### Procedures for post-visit assessments

After T1 is complete, RAs communicate with participants via phone calls, texts, and emails to schedule the next two surveys at the clinic, online, or by phone. If participants gave RAs permission to leave voicemail messages, RAs leave messages explaining that “It’s time to complete the next step of the Women’s Health Study, please call ###-###-#### to make arrangements.” The RAs’ email addresses used for correspondence to schedule surveys and send online survey links lack features that identified the true purpose of the study (for example: healthy9@pitt.edu). Language used in the email titles and bodies is also general and pertains to the “Women’s Health Study.”

**Fig. 4:**
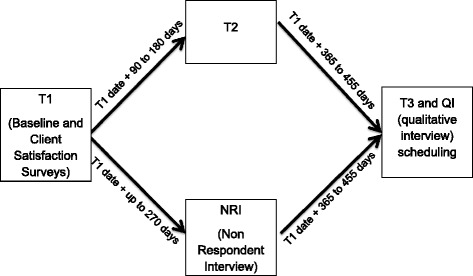
Interview schedule. For patients not completing T2, a Non-respondent interview is collected to enhance retention at T3

Participants become eligible to complete their follow up surveys at three months (T2) post baseline and again at 12 months (T3) post baseline (Fig. 4). RAs schedule times to meet participants back at the clinic to complete the ACASI surveys. If participants 18 years and older are unable to come to the clinic they are offered the option of completing the survey online via Survey Monkey or over the phone. If a participant cannot come to the clinic and is under 18 year old, they are offered to the option to complete the follow up surveys via phone.

If RAs are not able to contact a participant to schedule the T2 schedule within the three to 6 month window following baseline but regained contact thereafter, RAs offer the option of completing an interview about their experience with the study for $40 compensation. These interviews assess the barriers participants experienced such that they couldn’t complete T2 within the specified time frame. The most common barriers were not having time and not having access to a private computer to take the survey online. At the conclusion of this non-respondent interview (NRI), the interviewer worked with the participant to schedule her T3 survey.

The sub-sample of intervention participants over age 18 who complete T3 either in-person or by phone are asked whether they would be interested in learning about an optional study component. Interested participants complete a brief computerized screen to determine their eligibility for a narrative interview about their experiences receiving the intervention. The narrative interviews are intended for women who have ever experienced IPV or RC, as the intervention is likely to be most relevant for this subsample of women. Eligible women respond affirmatively on this screening survey to having ever experienced IPV or RC. The RA explains the interview would include sharing her relationship history as well as perspectives on receiving the intervention in the clinic. These interviews are conducted at the clinic at a time convenient for the woman, and she receives $50 to thank her for her time. Survey data are not connected to these anonymous narrative interviews.

At the conclusion of both T2 and T3 surveys, the same protocol for distributing gift cards, resource sheets, and safety cards is followed. If surveys are completed online or by phone, gift cards are mailed to participants with a generic return address that would not identify their participation in this study.

At the conclusion of T3 data collection, available FP medical records are reviewed for participants who had signed authorizations allowing the study team to do so (98 % of participants). RAs review visits spanning 12 months pre-baseline to 18 months post-baseline to record number and types of clinic visits, reasons for visits, pregnancy and STI testing and diagnoses, documentation of assessment for IPV and RC, and documentation of any disclosures of IPV or RC.

### Retention

Throughout the course of the study, research personnel use multiple methods to keep participants engaged in the study. One method is to call approximately 1–2 times per month to verify contact information. In addition participants are contacted to set up follow up surveys approximately 4–6 weeks before their “due” date, and then are reminded the day before and on the day of appointments. If a participant misses their window for participation in the T2 survey (beyond six months after baseline), they are contacted for a non-respondent interview (described above) where they are asked questions such as “We'd like to know what some of the reasons were that you were not able to do the survey?” and “What would have made it easier to come back to do the survey?” This information informs improvements to the protocol to add additional funds for bus fare on women’s gift cards, as transportation (and not having enough funds for gas) was a common reason stated for not being to make it to appointments.

### Power calculations and sample size

The planned recruitment of 3600 subjects from 25 clinics in 17 clusters was designed to achieve 80 % power to detect clinically significant effects of the intervention (under two-sided testing at alpha = 0.05) for each of the specific hypotheses, after making appropriately cautious adjustments for loss to follow-up, variance inflation arising from cluster randomization, repeated follow-up measurements (of most outcomes), the small number of denominator degrees of freedom available when 17 clusters are used to assess the effect of the intervention, and for the restriction of the Aim 2 analyses to subjects who reported recent IPV or RC victimization at baseline. Reduction of RC and IPV by 50 % (*OR* = 0.50) in both the full sample and subset experiencing IPV/RC at baseline were specified as clinically significant effects. We also specified that reduction in recent unintended pregnancy at the 12-month follow-up by 43 % (*OR* = 0.57) in the full sample would represent a meaningful clinical effect. Clinically significant effects were also specified for other primary and secondary outcomes, corresponding to intervention effect sizes of 0.20 standard deviations and 0.35 standard deviations for Aim 1 and Aim 2 analyses, respectively.

Calculations were guided by results from our pilot study, [26] allowing us to anticipate that the prevalence of recent reproductive coercion in the control group would be 10 % in the Aim 1 sample and 26 % in the Aim 2 sample and we powered the study to detect reduced prevalence of this outcome in the intervention arm of 5.3 % and 14.9 % in the Aim 1 and Aim 2 analyses, respectively. Similarly, assuming the prevalence of recent unintended pregnancy is 20 % in the control arm at the 12-month follow-up (from pilot data), our study was powered to detect a prevalence reduced to 12.5 % in the intervention arm in the Aim 1 analysis.

Based on results from the pilot study, we anticipated retaining at least 80 % of baseline subjects for the first follow-up assessment. We further assumed that at least 75 % of the subjects from the first follow up would also complete the second follow-up assessment, that approximately 17 % of subjects would report recent IPV victimization at baseline. To account for cluster randomization design effects, we assumed that in our planned regression analysis the residual within-person and within-clinic correlations would be no higher than 25 % and 1 % respectively for key outcomes. The above conditions indicate that our planned sample size of 3600 patients and repeated measurements for most outcomes would result in analyses that would provide an effective sample size for Aim 1 analyses of at least 798 for assessing the unintended pregnancy outcome (at the 12-month follow-up) and at least 1000 for the other Aim 1 outcomes (where observations at both follow-up occasions would be analyzed jointly – i.e., pooled T2 and T3 data). For the Aim 2, we anticipated an effective sample size of at least 424 observations for the other key outcomes in Aim 2 (where assessments from both follow-up occasions would be analyzed jointly). If attained, these effective sample sizes, based on prudently cautious assumptions, ensure at least 80 % power to detect clinically significant effects for the main outcomes in the analyses for Aims 1 and 2.

### Data safety and monitoring plan

The ARCHES intervention is focused on providing education, relevant counseling and support for females ages 16 to 29 years seeking care in family planning clinics. The pilot study enrolled over 1200 women, allowing for careful process evaluation to examine the impact of the intervention on client and clinic provider safety. To our collaborative team’s knowledge, no adverse events emerged in the course of the pilot implementation. We ensured that the intervention clinics received specific training on mandated reporting requirements and how to conduct this reporting in as safe and client-centered way as possible as an integral part of the intervention. In addition to usual precautions regarding protecting client privacy and confidentiality, we also obtained a Certificate of Confidentiality to protect the research data from subpoena. No requests for data occurred during the pilot study. Despite the low risk associated with this behavioral intervention, we organized an internal data safety and monitoring plan as well as an external data safety and monitoring board. The Internal Data Safety and Monitoring board is comprised of the two Co-PIs (Miller and Silverman) and the trainer from Futures Without Violence (Levenson). The internal review board meets by conference call once monthly and can confer on an as needed basis should adverse or difficult events occur. The functions of this internal DSM board are to:

- Systematically review assessment materials to ensure that assessment was conducted appropriately and that participants disclosing abuse or violence receive appropriate connection to violence-related services and that mandated reports are made by clinicians when appropriate.
- Systematically review notes from research assistants to ensure that participants experiencing distress were connected directly with the on-site health care provider, receiving educational materials, and being referred appropriately; this included ensuring that all research assistants documented asking each participant about emotional distress after completion of the survey.
- Conduct on-going preliminary data analyses to evaluate the potential impact of the intervention on specified outcomes of interest (knowledge, self-efficacy, behaviors). In the unlikely event that the intervention appeared to decrease the likelihood of help seeking or increases the distress of participants, the study protocol would be reexamined and modified appropriately.
- Monitor staff performance with regard to protection of privacy, confidentiality, maintenance of secure databases, and study procedures designed to reduce the risk of distress and potential breaches of confidentiality.
- Ensure that the PI (Miller), or a designated qualified individual, is available by pager in case research staff needed to confer regarding participant symptoms.
- Review and report any adverse events associated with the study.

The current project involves monitoring activity across 25 clinics, a much larger undertaking than the pilot study, with follow up of clients up to 15 months from baseline. Given the complexity of this project, in addition to the internal monitoring noted above, we instituted an External Data Safety and Monitoring Board. This external monitoring group consisted of the Quality Assurance Director from Adagio Health, the Vice President of Operations from Planned Parenthood Western Pennsylvania, and Director of Health from Futures Without Violence. All three individuals are familiar with the details of the research protocol, but not intimately involved in the day to day implementation. They also have the authority to suspend the project should any challenges or adverse events arise, and to direct changes in clinic policies and research protocol should site-specific problems emerge. For example, should a client experience emotional distress and the research assistant notified designated clinic staff but the staff is not responsive, this would be reported immediately to the Internal DSMB as well as the External DSMB, and changes would be implemented before proceeding with any further data collection.

### Patient accrual and study flow

**Fig. 5:**
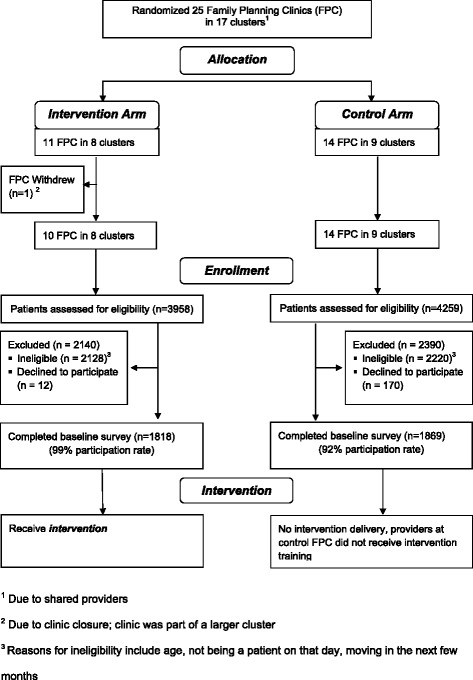
CONSORT flow diagram

25 clinics were combined into 17 clinic clusters for randomization, 8 intervention and 9 control clusters (Fig. 5). From intervention sites a total of 3958 women were approached, 2140 who were excluded either because they didn’t meet inclusion criteria or because they declined to participate, and from control clinics a total of 4259 were approached with 2390 being excluded leaving 3687 who were enrolled into the study and completed the baseline survey (Table 2).

**Table 2:** Demographics of total collected sample (*n* = 3687)

Characteristic	Total %^a^ (n)
Age	
16–20	37.7 (1387)
21–24	35.6 (1310)
25–29	26.8 (986)
Race	
White	80.1 (2951)
Black/African American	13.4 (492)
Hispanic/Latina	1.6 (59)
Multi-racial	3.0 (110)
Other	1.6 (59)
Education	
Less than 12^th^ grade	19.2 (707)
Finished high school	27.0 (995)
Some college	32.9 (1211)
Finished college or grad school	20.3 (748)
US Born	
Yes	98.2 (3615)
No	1.8 (65)
Relationship Status	
Single	32.1 (1181)
Dating more than one person	1.5 (56)
Dating one person/in a serious relationship	58.3 (2148)
Married	7.0 (259)
Married with more sex partners than husband	0.2 (7)
Ever had sexual intercourse	
Yes	97.9 (3604)
No	1.9 (69)
Sex of Sexual Partners^b^	
Men only	88.6 (3184)
Mostly men	7.8 (279)
Equally men and women	1.6 (58)
Mostly women	0.3 (12)
Women only	1.7 (62)

^a^%s may not equal 100 % due to small amounts of missing data

^b^Of those who report ever having had sexual intercourse

### Planned analytic approach

#### Overview

Following a sequence of preliminary analyses to characterize the sample participants and outcome measures, statistical methods for cluster-randomized experimental studies will be used to assess study hypotheses. The anticipated study data is multilevel, with baseline and follow-up data collected from patients, the units of analysis, who are nested within clinics, the units of randomization. The primary assessment of intervention effects will be based on intent-to-treat estimates. As-treated effect parameters will be estimated in secondary analyses and reported as exploratory. Following preliminary phases of descriptive, bivariate and multivariate analyses to characterize the sample and to refine and characterize scale-based outcome measures, estimates and hypothesis testing will be conducted in a multilevel generalized linear mixed (regression) modeling framework that will specify within-clinic and within-patient cluster effects. Regression models will include a parsimonious set of covariates to adjust for the restricted stratified randomization of clinics, to reduce the potential for confounding arising from between-arm imbalances in patient and clinic characteristics, and to increase the precision of intervention effects, with the specification of link and variance functions tailored to the type of outcome variable (e.g., logistic links for binary outcomes).

Two types of biases will be specifically explored using statistical testing. Participation bias will be assessed by comparison of age, race/ethnicity, and other demographics of clients participating in the study with that of the demographics of the overall family planning clinic clients (at participating sites) during the period of data collection, with significant differences noted as potential threats to generalizability. Descriptive statistics and bivariate and multivariate analyses will characterize patient demographics and compare groups with respect to key baseline measures. Variables differing across treatment groups at the p < .20 level will be included as covariates in subsequent adjusted outcome analyses. A similar set of analyses will be conducted to compare those who complete all three visits to those who do not to assess attrition bias. If between-arm differences in attrition are found, this potential validity threat will be noted.

#### Outcome analyses

We will use generalized linear mixed effects models for evaluating the effect of the intervention on follow-up outcomes. Separate models will be created for each outcome, and predictors will include a parsimonious set of important covariates, including potential confounders identified in preliminary analyses, the baseline measure of the outcome (when available), and a clinic-level binary indicator for whether the patient was in a treatment (vs. control) clinic. Random effects will be specified to account for the nesting of patients within clinic and, where appropriate, for repeated follow-up measurements. Recognition of sexual coercion, reproductive coercion, and physical and sexual partner violence baseline measures will be available for all time points and thus baseline measures will be included as covariates in the respective models to improve the precision of our estimated intervention effects. Within-clinic and within-patient cluster effects will be specified as random effects in the models.

For Aim 2 hypotheses, the analysis will be restricted to the patients who report at baseline having recently experienced physical or sexual partner violence or reproductive coercion.

Following the definitive assessments of key study hypotheses, a series of exploratory analyses will be conducted. In particular, the potential for differential impact of the intervention based on patient characteristics will be evaluated, with a view toward identifying factors (e.g., race/ethnicity, age, immigrant status, involvement in commercial sex) that may facilitate or impede the efficacy of the intervention, and to identify patient subgroups most receptive to the intervention or that may require further assistance.

For each outcome, we will use all eligible records at both follow-up visits to model timepoint-specific intervention effects, with the first model specifying (TIMEPOINT x INTERVENTION) interaction terms to permit testing for homogeneity, using Wald-tests with a statistical significance threshold of 0.05. For outcomes without statistically significantly heterogeneous intervention effects, the primary assessment of the intervention will involve the overall main effect of the intervention (versus the control), pooled across both follow-up occasions. Otherwise, timepoint specific contrasts will be used for the primary assessment of the intervention on the outcome. Each outcome will be assessed with a two-tailed alpha = 0.05. No adjustments for multiple testing will be made, as we feel that each of these hypotheses is of independent scientific interest. Graphical and analytical methods will be used to examine model fit and to evaluate assumptions used to justify inferences.

#### Missing data

The eligible population for these analyses is restricted to measurements from women who report (i.e., at the given timepoint) having heterosexual sexual intercourse (vaginal, oral, or anal). At each timepoint, this eligibility criterion was reassessed and further survey data necessary for defining outcome variables at that timepoint were collected only when this criterion was met. For women with follow-up data for whom the baseline survey did not collect responses needed to compute baseline covariates to be used in the mixed-effects models, baseline covariate values will be imputed.

In addition, for intermittently missing values (e.g., when the respondent selected “don’t know” or “refuse to answer”) for items in eligible follow-up visits, values will be imputed using multiple imputation (Markov Chain Monte Carlo algorithm) procedures in SAS PROC MI and PROC MIANALYSE for the imputation and analyses steps, respectively.

## Discussion

This community-partnered study tests a brief intervention to reduce risk for intimate partner violence (IPV) and reproductive coercion, with the goal of reducing unintended pregnancy among young, medically-underserved women attending family planning (FP) clinics. IPV is experienced by a disproportionately large number of women seeking care in FP clinics, and experiences of IPV have consistently been observed to relate to higher risk for unintended pregnancy. A team of researchers, clinicians, advocates, and FP clients, collaborated to develop the ARCHES intervention that integrates universal education and assessment for *reproductive coercion* (a recently identified phenomenon strongly associated with IPV and unintended pregnancy) and related harm reduction strategies into standard FP care. Delivered via the existing clinic structure of family planning counseling and clinical services, this intervention is not only innovative, but sustainable and replicable. Thus, if found effective, this intervention may be scaled-up at relatively low cost to address the intertwined major public health concerns of unintended pregnancy and IPV.

Our pilot study confirmed the acceptability and feasibility of this intervention, and demonstrated a significant reduction in pregnancy coercion at follow-up among women reporting recent IPV. This larger-scale cluster-randomized controlled trial engaged a greater number of clinics (*N* = 17 clusters) and participants (*N* = 3687) for a longer trial duration (12 months) to achieve sufficient statistical power and length of time post-baseline to assess for reductions in key outcomes - IPV victimization, reproductive coercion, and unintended pregnancy.

In conducting the trial, we encountered several challenges and limitations:

- While we attempted to enroll a large number of clinics, due to the overlap of staff and clinicians across sites, 25 clinics resulted in only 17 clusters.
- After randomization, one of the intervention clinics closed before we could enroll participants (one of two clinics in a rural cluster), resulting in 24 clinics. After T1 data collection was completed, another intervention clinic (urban) closed. Because of the research team’s system for obtaining multiple ways to contact participants, many of the participants from that clinic could be found and could complete the survey at another clinic location in downtown Pittsburgh (or via email or phone).
- While the survey was available in Spanish, almost all the participants were English speaking, predominantly White, with very small numbers of non-African American minorities in this sample, limiting the generalizability of study findings to more diverse settings across the U.S.

In summary, the results of this RCT will address the question of whether brief universal IPV/RC education and counseling intervention for IPV and RC in family planning clinic settings can help to reduce risk for IPV and RC and, in turn, decrease unintended pregnancy. The trial will also examine how this intervention may affect women who have recently experienced IPV or RC, with attention to their use of IPV resources and disclosure to health care providers, in addition to the overall primary and secondary outcomes. Additional process evaluation including interviews with clients and providers will provide information on barriers, challenges, and facilitators of intervention implementation. This study will contribute to the growing evidence base on the utility of brief educational and counseling interventions in clinical settings to address IPV an RC; additionally, lessons learned from this clinical intervention implementation may guide the design and implementation of similar interventions to increase overall health and well-being.

## Abbreviations

PCP: Primary Care Provider
ARCHES: Addressing Reproductive Coercion in Health Settings (Reproductive Coercion and Partner Violence Intervention)
RC: Reproductive coercion
FP: Family planning
IPV: Intimate partner violence
SA: Sexual assault
RCT: Randomized controlled trial
RA: Research assistant
NRI: Nonrespondent interview
QI: Qualitative interview
ACASI: Audio computer-assisted self-interview
